# Recent Advances in the Direct Electron Transfer-Enabled Enzymatic Fuel Cells

**DOI:** 10.3389/fchem.2020.620153

**Published:** 2021-02-10

**Authors:** Sooyoun Yu, Nosang V. Myung

**Affiliations:** Department of Chemical and Biomolecular Engineering, University of Notre Dame, Notre Dame, IN, United States

**Keywords:** enzymatic fuel cell, direct electron transfer, glucose oxidase, nanostructure, biocatalyst

## Abstract

Direct electron transfer (DET), which requires no mediator to shuttle electrons from enzyme active site to the electrode surface, minimizes complexity caused by the mediator and can further enable miniaturization for biocompatible and implantable devices. However, because the redox cofactors are typically deeply embedded in the protein matrix of the enzymes, electrons generated from oxidation reaction cannot easily transfer to the electrode surface. In this review, methods to improve the DET rate for enhancement of enzymatic fuel cell performances are summarized, with a focus on the more recent works (past 10 years). Finally, progress on the application of DET-enabled EFC to some biomedical and implantable devices are reported.

## Introduction

Since its first demonstration of concept by Yahiro et al. (1964), enzymatic fuel cell (EFC) has gained much research interest as one of the environmentally friendly and renewable source of power generation. Utilizing isolated enzymes from microorganisms as biocatalysts at either or both of electrodes, EFC is particularly attractive as the substrate specificity of the biocatalysts essentially removes the need for compartmentalization of each electrode, allowing for wider applications *via* miniaturization. The biocatalyst at the anode catalyzes the oxidation reaction of a fuel, from which electrons are released and then transferred to the electrode surface. The electrons then travel through the circuit to the cathode, where they are consumed in the reduction reaction of an oxidant, typically oxygen to produce water as byproduct. In an EFC, there are two possible electron transport mechanisms from the redox center of the biocatalyst at the anode (i.e., bioanodic enzyme) to the electrode surface: direct electron transfer (DET) and mediated electron transfer (MET). When an electron generated from oxidation catalyzed by the redox center of a bioanodic enzyme travels directly to the electrode surface and is collected as current, the enzyme is known to undergo DET; when an additional component is utilized between the enzymatic catalyst and electrode surface to act as a mediator to shuttle the electron, it is referred to as MET. Early works with EFC typically involved electron mediators, such as hydroquinone, benzoquinone, and ferricyanide salt to obtain current (Hunger et al., 1966; Davis and Yarbrough, 1967). Though first report of DET may date back to as early as 1972 (Betso et al., 1972), it was not until 1978 when works by Berezin et al. pioneered the DET mechanism to collect current with a laccase (Lc) directly adsorbed onto graphite electrodes (Berezin et al., 1978).

Despite over a half of a century worth of research, there is yet to be a consensus on whether DET or MET surpasses one another in terms of EFC performance. On one hand, MET-enabled EFC theoretically provides higher current and power density as the mediator would minimize the number of electrons that fail to reach the electrode due to its small tunneling distance (~10 Å); on the other hand, DET-enabled EFC offers simpler configuration and can bypass potential toxicity or low stability of some mediators (Mazurenko et al., 2017a; Mani et al., 2018). Furthermore, not using a mediator means the redox enzymes can operate at a potential close to their natural standard redox potentials, leading to lower chances of interfering reactions as well as higher open-circuit potential and thus higher power density (Kawai et al., 2014).

When evaluating the performance of EFC, several properties are commonly characterized or quantified: power density, current density, the amount of decay in power density over time, and open-circuit potential (OCP). The power and current density and the OCP correlate to the overall power output by the EFC, while the time-dependent decay of the power density can be translated into the stability of the EFC. While DET seems like a more favorable method of electron transfer to optimize the EFC performance, the small electron tunneling distance greatly limits the type of oxidoreductases that can be used for this configuration. Typically, the redox center is deeply embedded within a protein matrix, the size of which often exceeds 10 Å. In fact, only about a 100 of 1,700 known oxidoreductase enzymes can facilitate DET (Shleev et al., 2016). In an effort to not only increase this number but also enhance the fuel cell performance utilizing DET, a number of components of EFC could be improved: protein engineering to increase the efficiency of the direct electron transfer; immobilization of the biocatalysts on the electrode to decrease the tunneling distance and enhance the stability; and use of functional nanomaterials as electrodes to maximize enzyme loading while minimizing IR drop and tunneling distance for efficient charge transfer.

Herein, methods to enhance the performance of DET-enabled EFC that have recently been popular are summarized, and the outlook on these EFCs, including their applications are presented.

## Biocatalyst Engineering

One of the major drawbacks of using native enzymes as biocatalysts for EFCs is that some redox cofactors, molecules that change their oxidation state during catalytic redox reaction of the substrate, are deeply embedded inside the enzyme, and thus it is difficult for the electrons generated from the oxidation reaction to transfer successfully to the electrode surface (Hecht et al., 1993). For many enzymes, this means the electrons must be able to travel far beyond their 10-Å limit to reach the electrode, and most, if not all, of the electrons are not collected as current without any modification on the enzymes.

### Enzyme Choice and Protein Engineering

Glucose oxidase (GOx), one of the most extensively studied oxidoreductase enzyme for catalysis of glucose oxidation, is well-known for its flavin adenine dinucleotide (FAD) cofactor embedded within the protein matrix as far as 15–26 Å from the surface (Luong et al., 2017). Due to the large depth in which the FAD is located, some works have claimed that native GOx does not undergo DET at all (Wilson, 2016; Bartlett and Al-lolage, 2018). In these works, the redox peaks at E^0^ = −0.46 V (vs. Ag/AgCl) famously known to represent the GOx activity by the redox of FAD/FADH_2_ cofactor were argued to be inaccurate, as their electroanalytical methods with various control experiments suggested the redox peaks were due to the enzymatic activity of the FAD cofactors that have denatured from GOx itself, rather than the electroactivity.

Despite these claims, efforts toward DET-enabled GOx-based EFC have continued. Furthermore, other enzyme catalysts for both anode and cathode have been engineered and utilized for enhancement of EFC performance. This section describes various methods to modify or engineer enzymes for increasing the stability of enzyme immobilization and decreasing enzyme-to-electrode distance, thereby increasing the chance of DET for higher power density.

One of the drawbacks of using native GOx as the anodic biocatalyst is its sensitivity to oxygen. In addition to its primary substrate, glucose, GOx also interacts with oxygen as a natural electron acceptor and catalyzes its reduction to hydrogen peroxide. This can not only result in a lower coulombic efficiency, but also affect the cathode by depleting the available oxygen to be reduced (Navaee and Salimi, 2018).

**Figure 1:**
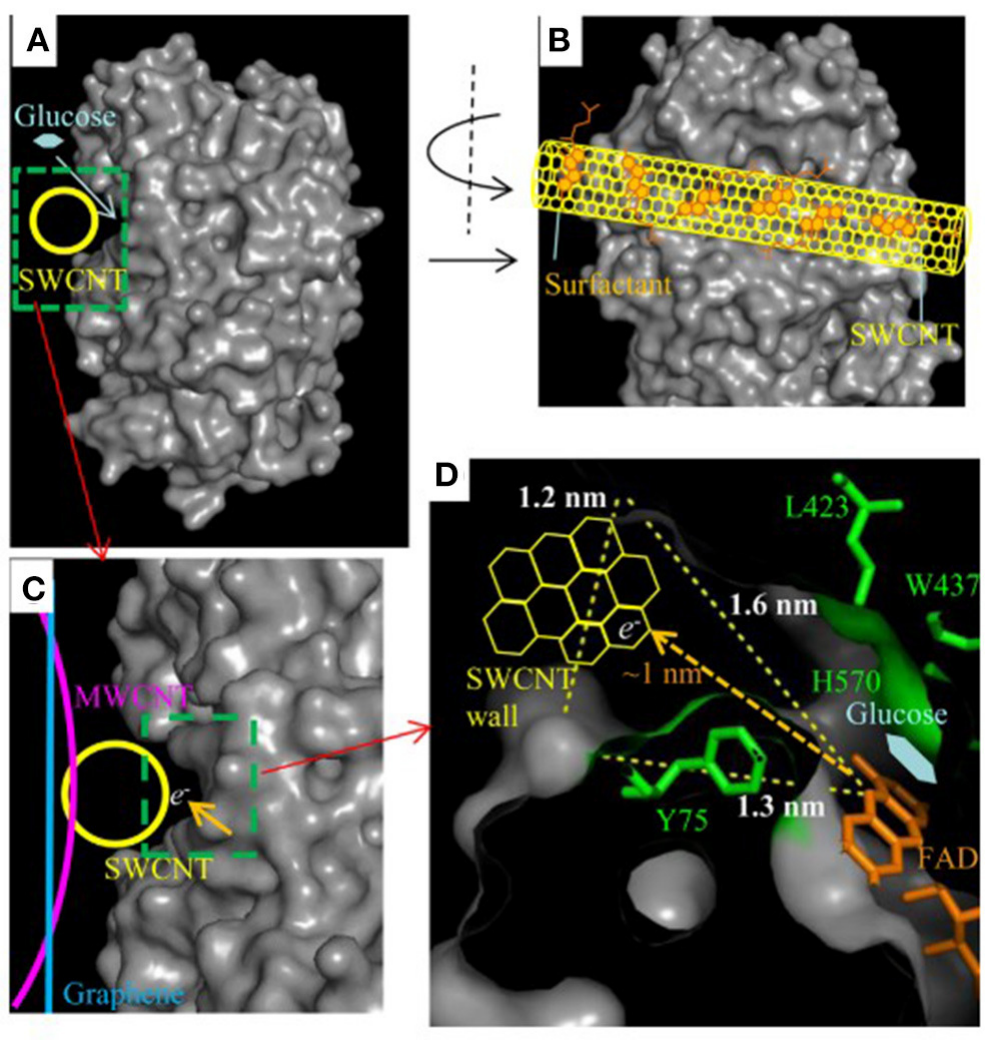
Schematic of possible DET route between FAD-GDH and debundled SWNT. **(A)** and **(B)** show possible location of debundled SWNT in the indentation of FAD-GDH; **(C)** compares the locations of MWNT, graphene, and debundled SWNT with respect to the FAD-GDH; **(D)** visualizes the reduced distance for electrons to travel from the FAD cofactor to the debundled SWNT (Muguruma et al., 2017).

As one of the alternatives, FAD-dependent glucose dehydrogenase (referred to as FAD-GDH) has been suggested as a promising enzyme. GDH is available with three different cofactors—pyrroloquinoline quinone (PQQ), nicotine adenine dinucleotide (NAD), and FAD. Though GDH based on all three cofactors have been utilized as anodic enzyme catalyst for EFC applications (Saleh et al., 2011; Schubart et al., 2012; Scherbahn et al., 2014), the low substrate selectivity and poor thermal stability of PQQ-dependent GDH (Aiba et al., 2015) and denaturing of NAD cofactor suggest there are room for improvement for them to be stronger candidates for glucose-oxidizing enzyme for EFCs. FAD-GDH, on the other hand, have been steadily used as an oxygen-insensitive alternative, immobilized in various EFC setups. Desriani et al. demonstrated FAD-GDH-based enzymatic fuel cell by casting FAD-GDH/carbon ink mixture on carbon cloth to fabricate the bioanode (Desriani et al., 2010). Combined with the cathode functionalized with bilirubin oxidase (BOD), the EFC produced up to 9.3 μW/cm^2^ power density with cellobiose as substrate. Muguruma et al. employed debundled single-walled carbon nanotubes (SWNTs), which were small enough in diameter (1.2 nm) to be plugged into the grooves of FAD-GDH to minimize the distance between enzyme cofactor and the electrode. Glucose concentration-dependent current response was only observed when debundled SWNTs were utilized as opposed to SWNT aggregates or multi-walled carbon nanotubes (MWNTs), despite the oxygen insensitivity of FAD-GDH (Figure 1) (Muguruma et al., 2017). Lee et al. studied the electrochemical behavior of FAD-GDH *via* chronoamperometry. Not only was DET achieved, but also the distance between enzyme cofactor and electrode surface was controlled by different self-assembly monolayers (SAM) to show its significance in enhancing the current response (Lee et al., 2018). Furthermore, the chemisorption between the thiol residue of SAM and gold electrode, combined with the covalent bond between the amino groups of the FAD-GDH and succinimide groups of SAM, strengthened the stability of enzyme immobilization on the electrode surface for more efficient DET.

Another method to overcome the weak DET rate by GOx is genetic modification for ready functionalization with metallic nanomaterials or more intricate structure with supporting materials. Prévoteau et al. demonstrated that deglycosylated glucose oxidase exhibited more negative surface charge than native GOx, which allowed for stronger electrostatic interaction with positively charged hydrogels used to immobilize the enzymes (Prévoteau et al., 2010). Electrodes functionalized with deglycosylated GOx showed higher current density to fixed amount of glucose than native GOx, which was attributed to the smaller enzyme-electrode distance and higher enzyme loading due to stronger attraction toward the hydrogel and thus the electrode surface. Other enzymes such as cellobiose dehydrogenase (CDH) was also deglycosylated to show up to 65% higher current response in the presence of substrate than the glycosylated enzymes (Ortiz et al., 2012). One of the advantages that contributed to this increase was the smaller hydrodynamic radius of the deglycosylated CDH, which allowed for higher amount of enzymes to be packed on the electrode surface. Holland et al. made direct mutations at various locations of GOx to add a cysteine side chain, which revealed a thiol group at a distance from 14 to 29 Å from the FAD cofactor. The thiol group attached to the GOx readily bound to gold nanoparticles, which facilitated direct electron transfer when functionalized onto electrode surface (Holland et al., 2011). Electroanalytical methods on electrodes modified with five mutated GOx showed only the enzyme with the thiol group closest to the FAD exhibited electroactivity, reinforcing the significance of minimizing enzyme-to-electrode distance for efficient DET. Other enzymatic catalysts such as fructose dehydrogenase (Hibino et al., 2017; Kaida et al., 2019), laccase (Lalaoui et al., 2016a), and pyranose 2-oxidase (Spadiut et al., 2009) have been modified for more stable immobilization as well as enhanced enzymatic activity.

### Orientation of Enzymes

Oxidoreductase enzymes are relatively large and measure few nanometers in diameter; the redox center is typically embedded within the protein matrix, at times tens of angstroms from the surface of the enzyme, which is well over the maximum electron tunneling distance of up to 20 Å (Moser et al., 1992). Because of this, it is essential to achieve favorable orientation of the enzyme when immobilizing on the electrode; that is, in a way that the redox cofactor is closest to the electrode surface (Lopez et al., 2018). The difficulties associated with obtaining such orientation, parameters that affect the orientation, and the electrode properties that are affected by the enzyme orientation are reviewed in great detail by Hitaishi et al. (2018). Thus, in this review, several recent works that demonstrated fine tuning of enzyme orientation by protein and electrode engineering for more efficient direct electron transfer are presented.

**Figure 2:**
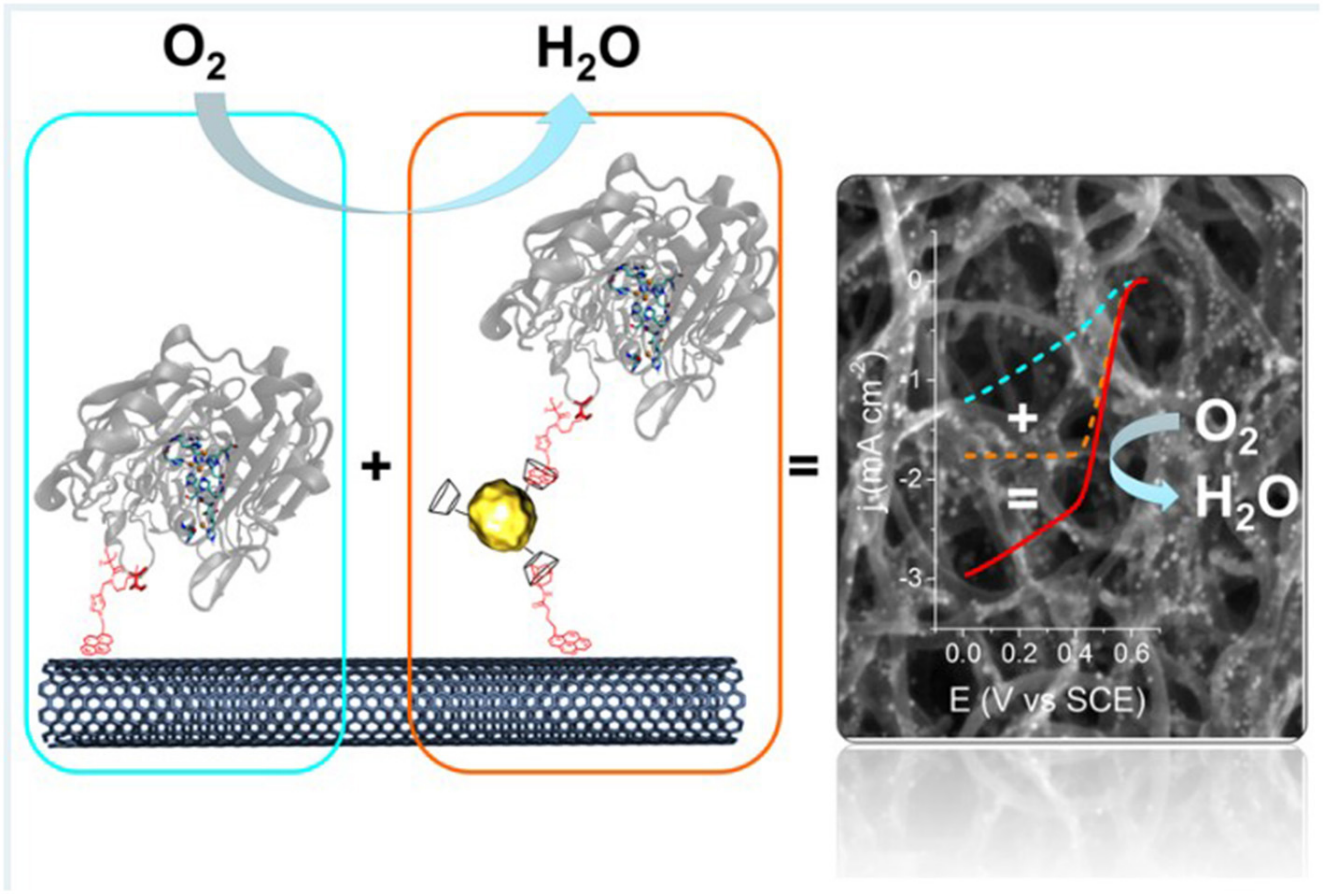
Schematic representing direct bioelectrocatalysis of pyrene-modified laccase immobilized by β-cyclodextrin-modified gold nanoparticles bound to carbon nanotube (Lalaoui et al., 2016b).

The general idea behind achieving good orientation for DET is to promote electrostatic interaction or covalent binding between the enzyme and the electrode surface so that the cofactor is located at a compatible distance from the electrode for electron transfer. For example, gold nanoparticles immobilized on highly oriented graphite electrode were functionalized with aminophenyl groups, which allowed for covalent binding between the nanoparticles and laccase enzyme. Two-step immobilization was proposed for this work: (i) the amino groups on the gold nanoparticles reacted with the oxidized sugar residues on the Lc, while (ii) amide bonds were formed between carboxylic groups of the enzyme and amino groups on the graphite electrode (Gutiérrez-Sánchez et al., 2012). Lalaoui et al. modified a specific location of laccase (Lc) with a pyrene molecule near the T1 copper redox center of the enzyme, so that when the pyrene group on the Lc bound to the CNT-bound gold nanoparticle, the redox center was closest to the electrode surface to maximize the current density output (Figure 2) (Lalaoui et al., 2016a).

Ma et al. engineered seven different mutants of cellobiose dehydrogenase to vary the orientation at which the enzyme was immobilized on the electrode surface. All enzymes but the wild type one were covalently bound to either gold or glassy carbon electrode, which was confirmed by surface plasmon resonance and cyclic voltammetry. The orientation of the enzyme immobilized on the electrode surface affected the mobility of the cytochrome domain, which moves between closed and open state to take the electron from the FAD cofactor to donate to the electrode. In the presence and absence of a mediator, they confirmed the enzyme cofactor-to-electrode distance controlled by the enzyme orientation greatly affected the DET/MET ratio (Ma et al., 2019). Though not employed in an enzymatic fuel cell setup, they electrochemically demonstrated this effect with increased current density in the presence of a substrate at a fixed concentration when the enzyme was in a DET-favorable orientation. Tasca et al. enhanced the direct electron transfer by modifying single-walled carbon nanotubes with *p*-aminobenzoic acid or *p*-phenylenediamine using aryl diazonium salts, and cellobiose dehydrogenase (CDH) was immobilized onto these surface-modified SWNTs as the bioanodic enzyme (Tasca et al., 2011). The functional groups provided a positively or negatively charged surface to increase the interaction between the enzyme and the electrode as well as to facilitate specific orientation of the enzyme. Tasca et al. further explained that at low pH (i.e., pH3.5), which exhibits high surface concentration of negatively charged amino acid residues, the protonated (i.e., positively charged) *p*-phenylenediamine could create a less electrostatically repulsive environment for CDH, thus enhancing the DET. This effort was continued by the same group, and a similar bioanodic setup was used to fabricate a third-generation biosensor to detect lactose (Tasca et al., 2013).

## Biocatalyst Immobilization Methods

Stability of enzymatic fuel cells can also be enhanced by securely immobilizing the catalyst on the electrode surface (Bahar, 2019). Without proper anchoring down or protection, biocatalysts could easily denature and lose their activity or desorb from the electrode surface. Several immobilization methods have been developed based on covalent bonding, affinity of biocatalysts, entrapment, crosslinking, and more. With the ample potential for miniaturization for applications in biomedical, biocompatible, and even implantable devices, it is critical to maximize the stability of the biocatalysts and thus of the device performance.

### Physisorption

**Figure 3:**
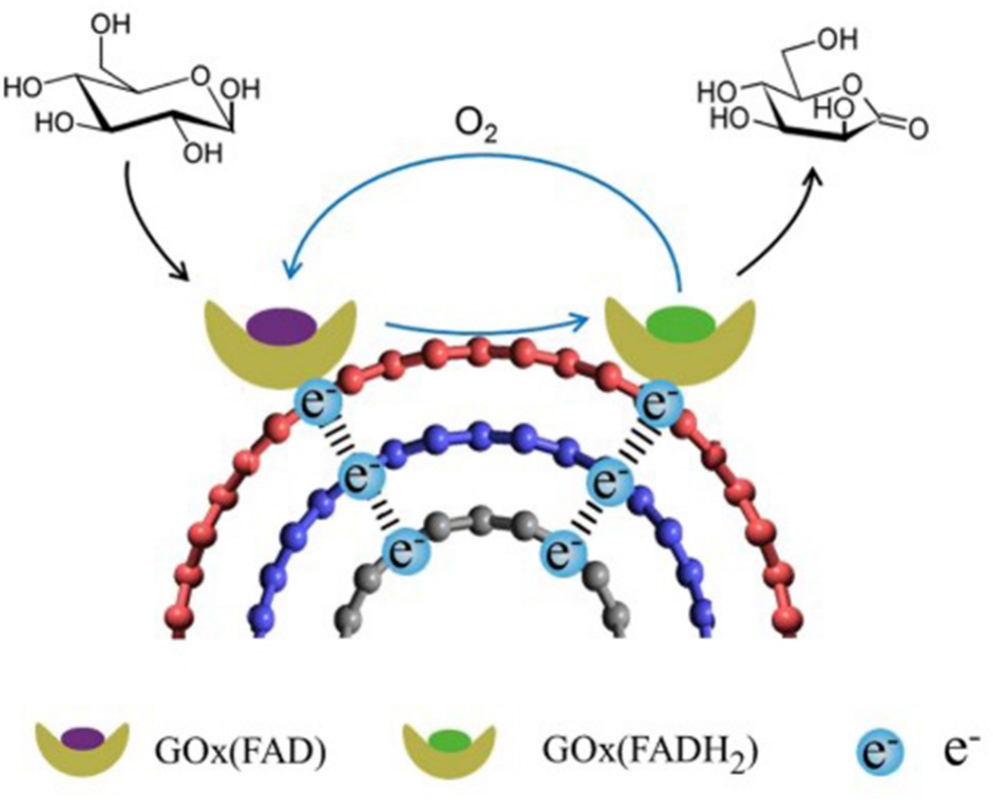
Schematic of proposed route of DET for GOx physisorbed with multi-walled carbon nanotubes (Liu et al., 2018) (https://pubs.acs.org/doi/full/10.1021/acsomega.7b01633. Further permissions related to the material should be directed to ACS).

Physisorption, typically done by dropcasting of enzyme solution onto the electrode surface followed by air-drying, is by far the simplest and cheapest method to fabricate enzyme electrodes. However, because the enzymes are immobilized by weak van der Waals forces or hydrophobic-hydrophilic interactions, they can easily desorb or leach off the electrode surface (Strack et al., 2013; Narváez Villarrubia et al., 2014). Because of this, physisorption is often avoided, and rather, novel methods of enzyme immobilization or development of composite electrode materials are sought, and thus physisorption is only briefly described in this review. However, there are still recent efforts to improve the stability of physisorbed enzyme catalysts, including composite materials to co-deposit with the enzyme solution. Das et al. utilized composite electrode consisting of reduced graphene oxide (rGO) and gold nanoparticles (AuNPs) to immobilize GOx, which showed higher electron transfer rate than when rGO or AuNPs were used individually. The improved performance of laboratory scale fuel cell built from this bioanode was attributed to the increase specific surface area and electronic conductivity of rGO combined with better attachment between GOx and AuNPs *via* sulfur-containing amino acids of the enzyme (Das et al., 2014). Liu et al. studied the effect of MWNTs on the DET and electroactivity of GOx by co-depositing carbon nanotubes of various numbers of layers for various electroanalytical methods, which suggested that the electrons generated from GOx was shuttled from outer to inner wall of the MWNTs (Figure 3) (Liu et al., 2018).

### Entrapment and Conducting Polymers

Organic molecules with metallic or semiconducting electrical properties called conducting polymers can be an effective agent to entrap the enzyme catalysts and help transfer electrons to the electrodes. In addition to these benefits, polypyrrole (Ppy) helps prevent some undesired reactions, explaining its continued use since its implementation in EFC setups in 1986 (Umaña and Waller, 1986). Recently, more complex bioanode setups with single- or multiwalled carbon nanotubes, nanocellulose, graphene, or various Ppy nanostructures, immobilizing a wide range of enzymes such as fructose dehydrogenase (Kizling et al., 2015, 2016), glucose oxidase (Kim et al., 2009; Min et al., 2010; Liu C. et al., 2011), and alcohol dehydrogenase (Gutiérrez-Domínguez et al., 2013). Many works suggested the enhanced power density was owed to the conductive polymer matrix allowing for proper orientation of the enzymes, good mass transport rates, and improved stability.

**Figure 4:**
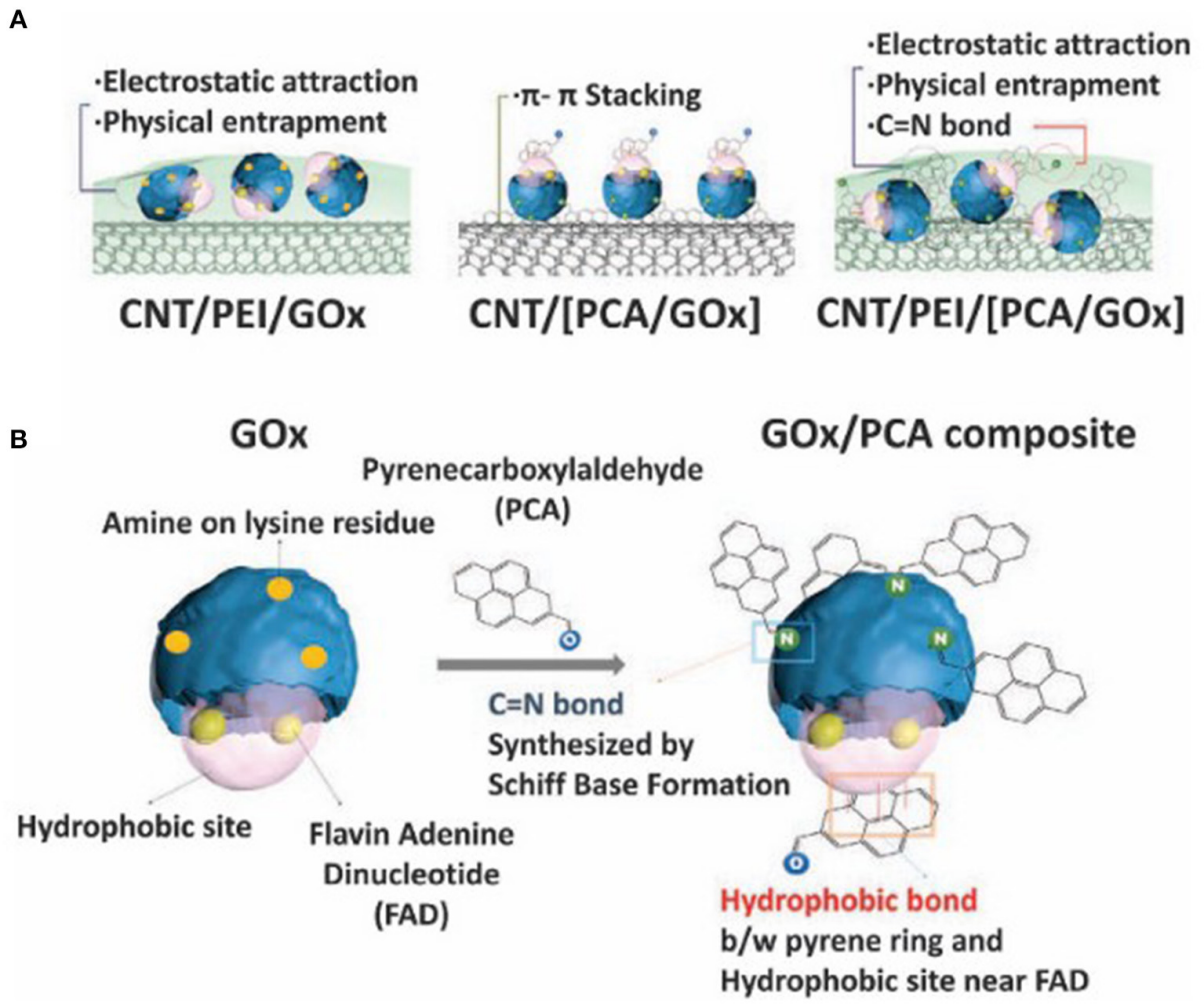
Schematic of **(A)** fabrication of various GOx-functionalized electrodes and their mechanism of immobilization; **(B)** comparison between native GOx and GOx/PCA composite (Christwardana et al., 2017).

Polyethyleneimine (PEI) is also widely used to immobilize enzymes while exhibiting water miscibility and high biocompatibility as well as offering various surface chemistries for stable binding to electrode surfaces or other nanomaterials (Figure 4) (Christwardana et al., 2017; Sapountzi et al., 2017; Tavahodi et al., 2017). PEI is typically used along with carbon nanotubes (Christwardana et al., 2016, 2017) or metallic nanoparticles (Zeng et al., 2015; Chung et al., 2017a; Christwardana et al., 2018) to increase the stability of enzyme immobilization.

Polyaniline (PANI), discovered over 150 years ago, only started gaining research interest until 1980's due to its high electrical conductivity, and was first demonstrated for its utility as enzymatic electrode in 1999 by immobilizing lactate dehydrogenase on electrochemically prepared PANI film (Gerard et al., 1999). Since then, PANI has been utilized as composite materials or prepared as nanofibers (Kim et al., 2011, 2014; Mishra et al., 2017), mainly due to its multifaceted functionality and biocompatibility (Yan et al., 2010). PANI can be directly electrochemically polymerized or functionalized onto various carbonaceous nanomaterials including graphene, graphene oxide, or carbon nanotubes for enhanced electrical conductivity and enzymatic activity (Schubart et al., 2012; Kashyap et al., 2015; Kumar et al., 2016; Kang et al., 2017).

### DNA as Scaffolds or Electron Acceptors

**Figure 5:**
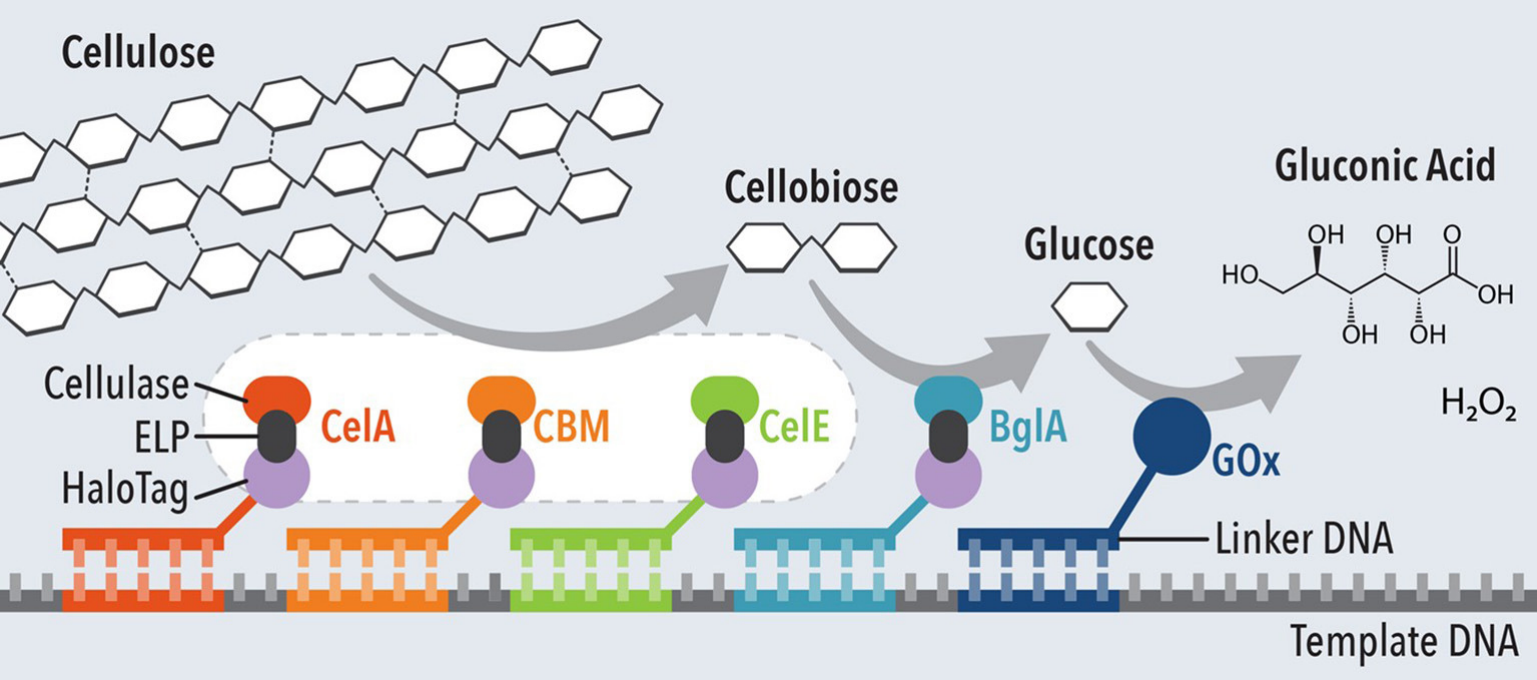
Schematic of five-enzyme cascade system for hydrolysis of cellulose and glucose oxidation. Adapted from Chen et al. (2017).

DNA has been employed as an effective method to immobilize single or multiple enzymes in a specific order for efficient cascade reactions. The terminals of the DNA can be modified for strong covalent bonds onto the electrode surface for stable anchoring of the enzyme catalysts. Xia et al. demonstrated a fully assembled methanol enzymatic fuel cell by immobilizing alcohol dehydrogenase and aldehyde dehydrogenase using zing-finger protein (Xia L. et al., 2017). The cascade reaction catalyzed by the two enzyme catalysts successfully hydrolyzed methanol to produce power density of 24.5 μW/cm^2^. DNA nanostructures have also been used to couple synergistic enzymatic reactions into a cascade system (Müller and Niemeyer, 2008; Conrado et al., 2012; Fu et al., 2012), up to five enzymes for sequential hydrolysis of cellulose (Figure 5) (Chen et al., 2017).

Other 3D structures utilizing DNA such as nano-chambers (Linko et al., 2015) and nanocages (Zhao et al., 2016) were fabricated for self-assembly of enzyme cascades by GOx and horseradish peroxidase (HRP), which showed enhanced activity compared to when the two enzymes were freely in solution. Chakraborty et al. studied an oxygen-reducing cathode catalyzed by bilirubin oxidase (BOD) (Chakraborty et al., 2015). They used DNA as a template to fabricate gold nanoclusters (AuNC), which enhanced the electron transfer, shown by 15 mV lower overpotential as well as 5.5 times higher current than when typical plasmonic gold nanoparticle was used in the same configuration. Furthermore, rolling circle amplification was utilized to assemble multiple copies of the enzyme catalysts for enhanced catalytic activity toward reactions that are otherwise impossible to achieve with a single enzyme (Wilner et al., 2009; Sun and Chen, 2016). Though some of these examples were not readily applied to EFC setups, it is worth noting their potential for future applications in EFC with enhanced catalytic activity and stability.

## Nanomaterial-Based Electrodes

The high surface area-to-volume ratio and variability of physical and chemical properties by precise control of morphologies have made nanomaterials attractive and superior to their bulk counterparts in numerous applications. Taking advantage of such properties to increase not only the enzyme loading but also protection around the enzyme catalysts can allow the nanomaterial-based EFC electrodes to increase stability and decrease the enzyme-to-electrode distance for more efficient direct electron transfer (Mazurenko et al., 2018).

### Carbonaceous Materials

Carbon-based nanomaterials exhibit high electrical conductivity and good mechanical properties, as well as various physical and electrical properties depending on the control of their morphologies. Various carbonaceous materials such as carbon fibers or papers (Xu and Minteer, 2012; Kuo et al., 2013), carbon black (Kamitaka et al., 2007; Gupta et al., 2011; Haneda et al., 2012; Xia et al., 2016a), carbon nanoparticles (Selloum et al., 2014), graphene (Chen et al., 2012; Campbell et al., 2015; Song et al., 2015), graphite (Tasca et al., 2015; Antiochia et al., 2019), and carbon nanotubes (Gao et al., 2010; Ciaccafava et al., 2012; Agnès et al., 2013) have been employed as EFC electrode materials. For example, buckypaper form of multiwalled carbon nanotubes (MWNTs) demonstrated excellent potential as enzymatic electrode material by 68-fold increase in current density from oxygen reduction reaction catalyzed by laccase compared to as-prepared agglomerates of MWNTs (Hussein et al., 2011). Hussein et al. attributed this significant enhancement to reduced diffusional mass transfer limitations and enhanced electrical conductivity when MWNTs were dispersed into a buckypaper form, which also exhibited highly mesoporous structures and good mechanical stability. Filip et al. demonstrated a low-cost biofuel cell by integrating carbon nanoparticle-nanotube composite-based bioanode and biocathode containing fructose dehydrogenase and bilirubin oxidase, respectively (Filip et al., 2013). In addition to combining two different carbon nanostructures to achieve high surface area as well as efficient interconnection for high electrical conductivity, this work by Filip et al. further attributed the high current density of the biocathode to the chitosan that acted as a “glue” to hold Ketjen Black-CNT composite, facilitating favorable orientation of the bilirubin oxidase for DET by electrostatic interaction between the positively charged chitosan and negatively charged enzyme as well as reducing charge transfer resistance and overpotential for oxygen reduction.

First discovered by Ijima et al. in 1991 (Iijima, 1991), carbon nanotubes (CNTs) now are one of the most widely used nanomaterials for fabrication of enzyme electrodes, largely due to high surface area-to-volume ratio and flexibility toward surface chemistry manipulation to enhance enzyme immobilization (Liu X. et al., 2011; Yan et al., 2011). For example, Zebda et al. demonstrated mediatorless glucose/oxygen biofuel cell based on GOx and Lc mechanically compressed with carbon nanotubes into bioanode and biocathode, respectively (Zebda et al., 2011). The high porosity and electrical conductivity of the CNT matrix contributed to good diffusion and electrical connection for the enzymes. Zebda et al. also suggested that the mechanical compression facilitated nanoscale proximity between the enzymes and the three-dimensional electrode surface, which attributed to the DET without any loss in enzymatic activity. In fact, power density of 1 mW/cm^2^ and open-circuit voltage of 0.95 V was largely retained for 1 month under physiological conditions. Though carbon nanotubes are typically physisorbed or compressed with enzyme catalyst of choice, followed by coating of semi-permeable polymer like Nafion to prevent desorption or leaching of the enzymes, more complex setups have been reported to enhance the immobilization of the enzyme as well as electron transfer by using electrode materials of higher surface area and electrical conductivity. Carbon-based electrode materials were chemically modified or doped for increased enzyme loading and stability of enzyme immobilization (Meredith et al., 2011; Karaśkiewicz et al., 2012; Wei et al., 2012; Giroud and Minteer, 2013), decorated with metallic nanomaterials for covalent bonding of enzymes and enhanced electron transfer (Naruse et al., 2011; Lalaoui et al., 2016a), or combined with various carbonaceous nanomaterials to form composite electrodes (Wu et al., 2013; Campbell et al., 2015; Escalona-Villalpando et al., 2018). For example, carbon nanotubes functionalized with naphthalene, an aromatic group toward which laccase exhibited affinity due to its hydrophobic pocket, were efficient electrode materials in not only ensuring electrical wiring between enzyme cofactor and the electrode surface but also increasing the amount of enzymes in favorable orientation for DET (Karaśkiewicz et al., 2012). Biofuel cell assembled with this electrode exhibited power density of 131 μW/cm^2^, 80% of which were retained after 24 h. Iron- and nitrogen-codoped carbon nanotubes were used by Ji et al. to enhance the overall catalytic activity of GOx-based bioanode by catalyzing oxidation reaction of hydrogen peroxide, a byproduct of glucose oxidation commonly known to inhibit enzyme activity (Ji et al., 2020). They also demonstrated their enzymatic fuel cell based on this electrode with power density of 63 μW/cm^2^, and ~80% of the bioanodic current density of 347.1 μA/cm^2^ was retained after 4 weeks.

### Porous Nanostructure

**Figure 6:**
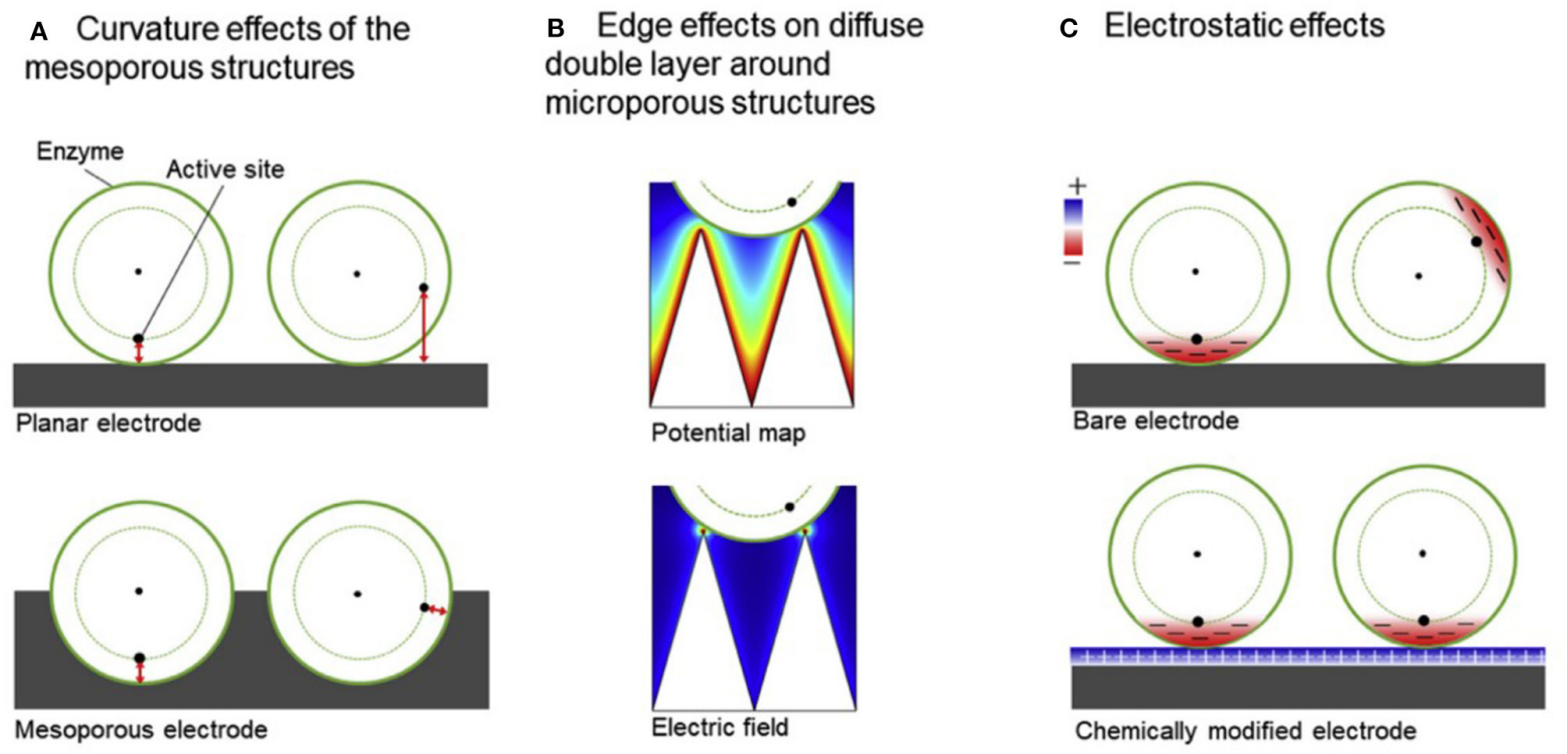
Schematics showing **(A)** curvature effect **(B)** edge effect **(C)** electrostatic effect that are taken into account for meso- and microporous structures (Sakai et al., 2018).

The ultra-large surface area-to-volume ratio with fine-tunable pore size, density, and overall nanostructure dimensions, porous nanostructures have shown to be excellent candidates for EFC electrode materials. Though enzymeless, catalytic glucose oxidation by Rong et al. described the high porosity of the polymer matrix employed around the gold nanoparticle catalyst provided size-selective protection against larger molecules (Rong et al., 2014). Similar to this work, enzymatic electrodes are also fabricated based on porous structures in order to protect the enzyme catalysts, while allowing more contact area to enhance the DET rate. The significance of mesoporous electrodes (i.e., containing pores of diameter between 2 and 50 nm) is supported by (i) the curvature effect (Sugimoto et al., 2016, 2017), in which the current density largely increases as pore diameter approaches that of a single enzyme; as well as (ii) the electrostatic interaction between the enzyme and the electrode, where the surface charge of the electrodes can promote the preferred orientation of the enzymes so that the distance between the enzyme active site and the electrode is minimized (Sugimoto et al., 2015; Lalaoui et al., 2016b; Xia et al., 2016b; Xia H. et al., 2017). With these in mind, meso- and microporous electrodes were fabricated based on glassy carbon electrodes modified with Ketjen Black and gold nanoparticles to improve the direct electron transfer kinetics of several redox enzymes such as bilirubin oxidase, hydrogenase, and formate dehydrogenase (Figure 6) (Sakai et al., 2018). Based on the electrochemical behavior of these electrodes characterized by cyclic voltammetry, combined with electrostatic charge distribution visualized by PyMOL, it was suggested that electrodes with controlled morphology such as mesoporous structure was a predominant factor in facilitating DET of the three enzymes. The mechanism of DET based on porous electrodes and the optimum porous nanomaterial for DET were mathematically modeled and experimentally validated by Do et al. (2014) and Mazurenko et al. (2017b) respectively.

Various porous materials were utilized as electrodes to be functionalized with enzyme catalysts. Wang et al. deposited single-walled carbon nanotube on gold-coated porous silicon substrates, onto which GOx and Lc were electrochemically immobilized to fabricate bioanode and biocathode, respectively. Enzymatic fuel cell built with these electrodes produced peak power density of 1.38 μW/cm^2^ for up to 24 h (Wang et al., 2009a). Though the power density output was not as high compared to similar works, it was important to note that both membrane- and mediator-free enzymatic fuel cell was demonstrated with glucose at a near-physiological concentration for potential in biomedical application. Improved peak power density and stability of up to 12 μW/cm^2^ and 48 h, respectively, were later demonstrated by the same setup and group later that year (Wang et al., 2009b). A work by du Toit and Di Lorenzo demonstrated the feasibility of highly porous gold electrodes for use in biofuel cell with GOx and laccase as anodic and cathodic catalysts, respectively, producing peak power density of 6 μW/cm^2^ (Du Toit and Di Lorenzo, 2014), a comparable value to other miniature EFCs (Beneyton et al., 2013; Falk et al., 2013). Their effort continued to develop a flow-through glucose/oxygen fuel cell based on highly porous gold electrodes for continuous power generation for up to 24 h (du Toit and Di Lorenzo, 2015). Salaj-Kosla et al. also utilized nanoporous gold electrodes to immobilize bilirubin oxidase by physisorption to produce catalytic current density of 0.8 mA/cm^2^ (Salaj-kosla et al., 2012). More recently, bioelectrocatalysis by bilirubin oxidase was further improved by immobilizing it onto porous gold (Takahashi et al., 2019a) and mesoporous carbon electrodes (Takahashi et al., 2019b), which was attributed to the appropriate pore size distribution as well as promotion of favorable orientation of the enzyme for direct electron transfer.

### Gold Nanoparticles

Due to their unique physical and electrical properties, gold nanoparticles (AuNPs) are among popular materials with which to functionalize EFC electrodes. In addition, the various facile synthesis protocols and size control techniques make AuNPs more attractive in enhancing the enzyme electrode performance. AuNPs themselves can be modified with functional groups such as thiols to covalently attach to electrode surface, improving the stability when the enzymes are adsorbed onto the AuNP functionalized electrodes, in which the AuNPs act as electronic bridges between the enzyme active site and the electrode surface. Monsalve et al. used a thiolated AuNPs of various sizes to covalently bind to a hydrogenase for bioanode fabrication, during which the smallest AuNPs were found to exhibit the highest surface area, leading to a 170-fold increase in current density from DET-based hydrogen oxidation compared to using unmodified bulk gold electrode (Monsalve et al., 2015). Combined with BOD-modified cathode, the as-fabricated EFC produced power density of up to 0.25 mW/cm^2^. Ratautas et al. fabricated a bioanode with AuNP modified with 4-aminothiophenol (4-ATP), which contained oxidized derivatives that allowed for stable immobilization of glucose dehydrogenase, and confirmed its mediator-free glucose oxidation electrochemically (Ratautas et al., 2016). Biocathodes based on AuNPs as electronic bridges for laccase were also fabricated by Kang et al.; laccase was immobilized onto naphthalenethiol-modified AuNPs, which promoted the electron transfer to the polyethyleneimine-carbon nanotube electrode (Kang S. et al., 2018). The high-surface area electrode also increased the enzyme loading, producing 13 μW/cm^2^ power density when put together into EFC. AuNPs were employed in both anode and cathode for sugar/oxygen EFC with cellobiose dehydrogenase and bilirubin oxidase as anodic and cathodic catalyst, respectively; the EFC showed improved performance than previously fabricated EFCs (i.e., power density of 15 μW/cm^2^ in buffer and 3 μW/cm^2^ in human blood), which was attributed to the use of 3D AuNP-modified electrodes (Wang et al., 2012). AuNPs are also used with other electrically conductive materials such as conducting polymers and carbon nanotubes to further enhance the electrical properties for more stable DET (Krikstolaityte et al., 2014; Tavahodi et al., 2017).

Recently, gold nanoparticle-carbon nanotube hybrid fibers were utilized as electrode materials for high-performance glucose/O_2_ enzymatic fuel cell (Kwon et al., 2019). GOx, the bioanodic enzyme of choice, and the gold nanoparticles were alternately assembled layer by layer to combine covalent and electrostatic bonding, leading to enhanced electron transfer and stability. This highly electrically conductive material allowed for fast electron transfer between the active site of GOx and the electrode surface, exhibited by the small redox peak separation of ~0.11 V. Furthermore, the assembled enzymatic fuel cell outputted power density and open-circuit voltage of 1.2 mW/cm^2^ and 0.98 V, respectively. In the presence of a low concentration of glucose to mimic the physiological conditions (i.e., 10 mmol/L glucose), the EFC still exhibited 0.6 mW/cm^2^ of power density and 0.72 V of open-circuit voltage, showing great promise in the biomedical applications. The same layer-by-layer assembly method was employed to fabricate GOx-coated metallic cotton fiber as the bioanode, exhibiting excellent electrical communication between the enzyme and the electrode for enhanced electron transfer (Kwon et al., 2018). Combined with the high conductivity of the cotton fibers (>2.1 × 10^4^ S/cm), the GOx/AuNP/metallic cotton fiber-based electrode showed an impressive power density of 3.7 mW/cm^2^ when assembled into EFC.

## Recent Performances of DET-Enabled Anodes, Cathodes, and EFCs

Combining methods discussed in the previous sections, recent studies have demonstrated enhanced performances of electrodes and enzymatic fuel cells operating in direct electron transfer. Studies worth highlighting are summarized in Table 1.

**Table 1:** Performances of electrodes and enzymatic fuel cells operating on direct electron transfer.

**Bioanodic enzyme/material**	**Biocathodic enzyme/material**	**Fuel/oxidant**	**Electrode/cell current density [μA/cm^**2**^]**	**Power density [μW/cm^**2**^]**	**OCP [V]**	**Lifetime [h]**	**% current or power density retained during lifetime**	**References**
CDH/SWNT/GC	-	Lactose/-	500	-	-	50	85	Tasca et al., 2011
CDH/graphite	-	Lactose/-	4.79	-	-	-	-	Ortiz et al., 2012
CDH/SWNT/GC	-	Lactose/-	500	-	-	288	50	Tasca et al., 2013
-	Lc/CNT/Ta	-/O_2_	840	-	-	168	75	Singh et al., 2020
GOx/hydroquinone/SWNT/Au	Lc/SWNT/Au	Glucose/O_2_	-	240	0.52	-	-	Bojórquez-Vázquez et al., 2018
Py_2_Ox/CAT/GC	Py_2_Ox/HRP/CNT-CMF-CC	H_2_/glucose	-	530	1.15	10	50	Ruff et al., 2018
GDH/PANI/AuNP/Au	-	Glucose/-	1,000	-	-	24	79	Gineityte et al., 2019
GOx/NQ/MWNT	HRP/MWNT	Glucose/H_2_O_2_	-	700	0.6	-	-	Abreu et al., 2018
GOx/TPA/PEI/CNT	Pt/C	Glucose/O_2_	78.6	1,620	-	672	75.8	Chung et al., 2017b
GOx/PANI/GC	Lc/PANI/GC	Glucose/O_2_	-	1,120	0.78	336	82.9	Kang Z. et al., 2018
FAD-GDH/Th-AuNP/CNT/GC	BOD/GR/CNT/GC	Glucose/O_2_	925	269	0.71	-	-	Navaee and Salimi, 2018
GOx/Naph-SH/AgNP/PEI/CNT	Pt/C	Glucose/O_2_	-	1,460	-	840	83	Christwardana et al., 2018
GOx/graphene	-	Glucose/O_2_	-	164	0.44	168	60	Babadi et al., 2019
GOx/PVP-RPPy/NiF	Lc/PVP-RPPy/NiF	Glucose/O_2_	-	350	1.16	336	82	Kang et al., 2019
Zn	BOD/MWNT/rGO/PG	-/O_2_	650	775	1.68	-	-	Torrinha et al., 2020
-	MoBOD/MWNT	-/O_2_	4,000	-	-	24	73	Gentil et al., 2018
GDH/GO/GC	Lc/AuNP/Au	Glucose/O_2_	1,100	400	0.86	576	93	Maleki et al., 2019

*CDH, cellobiose dehydrogenase; SWNT, single-walled carbon nanotube; GC, glassy carbon; GOx, glucose oxidase; Py_2_Ox, pyranose oxidase; CAT, catalase; GDH, glucose dehydrogenase; PANI, polyaniline; NP, nanoparticles; NQ, naphthoquinone; MWNT, multi-walled carbon nanotube; TPA, terephthalaldehyde; PEI, polyethyleneimine; CNT, carbon nanotube; FAD-GDH, flavin adenine dinucleotide-dependent GDH; Th, thionine; Naph-SH, naphthalene-thiol; PVP-RPPy, polyvinylpyrrolidone-rectangular polypyrrole; GO, graphene oxide; Lc, laccase; HRP, horseradish peroxidase; CMF, carbon microfibers; CC, carbon cloth; BOD, bilirubin oxidase; rGO, reduced raphene oxide; PG, pencil graphite; MoBOD, BOD from Magnaporthe orizae*.

Bioanode with one of the highest performances was demonstrated by Gineityte et al.; GDH was immobilized onto cysteamine-modified gold nanoparticles, which was tethered to polyaniline (Gineityte et al., 2019). The unique combination of conductive polymer directly electropolymerized on the electrode surface and positively charged monolayer of gold nanoparticles attributed to the enhanced electron transfer between the bioanodic enzyme and the electrode. This study further demonstrated the bioanode performance in human blood samples, in which the average current density was ~65% of that in blood-mimicking buffer solution. One of the highest power densities in an enzymatic fuel cell was reported by Chung et al., who utilized a two-step crosslinking method to first form a TPA/GOx composite and then immobilize onto PEI/CNT electrode (Chung et al., 2017b). The large power density of 1,620 μW/cm^2^ was attributed to the enhanced electron transfer due to electron delocalization by π conjugation as well as the enhanced stability of the enzyme. Because the GOx denaturation was reduced by the strong chemical bonds immobilizing the bioanodic enzyme on the electrode surface, the assembled EFC retained ~75% of its power density for 4 weeks, which highlighted the increased stability.

Some other methods that are interesting to note include extending the EFC lifetime by functionalizing magnetic nanoparticles with bioanodic enzymes to replenish the bioanode with a fresh batch of biocatalysts (Herkendell et al., 2019). Though power density of only 160 μW/cm^2^ was reported for this EFC, the idea of removing and reloading the biocatalysts by a magnetically assisted methodology was unique, which showed the EFC lifetime was extended from ~20 to 70 h.

## Recent Progress in Biomedical Application of DET-Enabled EFC

**Figure 7:**
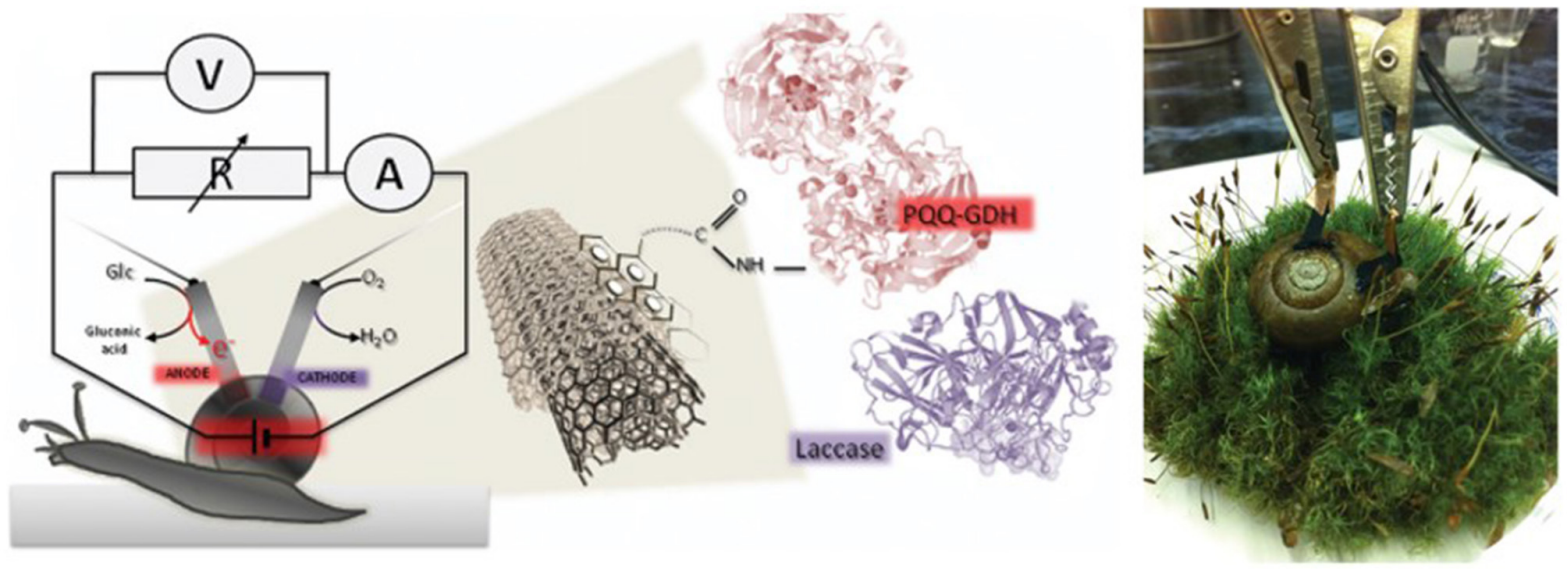
Schematic of enzymatic fuel cell using live snail. Figures are from work by Halámková et al. (2012).

Implantable devices powered by EFCs are increasingly attracting research attention since high substrate specificity of enzyme catalysts removes the need for compartments or membranes, allowing for miniaturization. Furthermore, use of biocompatible nanomaterials for electrodes has shown great potential in implantable EFC devices. Though mediated electron transfer-based EFCs have shown potential for implantable device applications earlier and consistently grown (Mano et al., 2003, 2004; Miyake et al., 2011; Sales et al., 2013), DET-enabled EFCs closely followed the trend. Starting from those fueled by clams (Szczupak et al., 2012) and lobsters (MacVittie et al., 2013), DET-driven EFCs were surgically implanted on an exposed rat tissue (Castorena-Gonzalez et al., 2013) and finally in the abdominal cavity of a rat to truly show the potential of biocompatible, implantable EFC to power devices, which was demonstrated by the powering of light-emitting diode (LED) and a digital thermometer (Zebda et al., 2013). To accomplish this, Zebda et al. wrapped the enzyme electrodes in silicone bags, followed by a dialysis bag filled with sterile solution and then an autoclaved commercial sleeve to avoid inflammation or toxicity issue with the rat tissue. Halámková et al. demonstrated a glucose/oxygen fuel cell based on PQQ-GDH noncovalently bound to carbon nanotubes and Lc as bioanodic and biocathodic catalyst, respectively, and generated maximum power density of 30 μW/cm^2^ and continuously operated as glucose was regenerated by the snail's feedings and relaxing (Figure 7) (Halámková et al., 2012).

The effort toward implantable EFC-powered devices for humans have also been consistent. Bollella et al. demonstrated a proof of concept with a FAD-based CDH and Lc as bioanodic and biocathodic enzyme catalyst, respectively, immobilized on a gold nanoparticle-functionalized graphene screen-printed electrode; the EFC with both enzyme catalysts co-immobilized on the same electrode showed power output of 1.10 μW/cm^2^ and open-circuit voltage of 0.41 V in real human saliva samples, hinting at a potential non-invasive autonomous biodevices (Bollella et al., 2018). DET-enabled EFCs also exhibited stable operation in other human liquids such as serum, saliva, and urine, ranging in power density of 12–18 μW/cm^2^ (Göbel et al., 2016). Operation of mediatorless EFC utilizing various enzymes such as PQQ-GDH and cellobiose dehydrogenase in real or synthetic human tear was observed, giving possibility of EFCs powering “smart” contact lenses (Falk et al., 2012; Reid et al., 2015). Other electrochemical devices for transdermal biosensing were reviewed by Tasca et al. (2019); minimally invasive diagnostic devices have gained some momentum, leading to microneedle-based biosensors penetrating the skin at the dermis (i.e., 1–2,000 μm) level to detect analytes in human body such as glucose, lactate, potassium ions, and glutamate.

Pankratov et al. utilized transparent, flexible substrate upon which CDH and BOx were immobilized was used to produce maximum of 0.6 μW/cm^2^ and maintain ~80% of the initial power density after 12 h of operation (Pankratov et al., 2015). The use of the transparent substrate and the minimal impact on the transparency upon enzyme immobilization, combined with relatively stable operation of the EFC, suggests its potential as a power source for smart contact lenses. The low power output of the implantable DET-enabled EFC in human physiological fluids [compared to ~40 μW/cm^2^ achieved by Milton et al. *via* mediated glucose oxidation in human serum at 21 °C (Milton et al., 2015)] was primarily attributed to the extremely low glucose concentration, but given that some cases of MET-based EFC was able to show higher power outputs, there are certainly more optimization and improvements needed before implementing DET-based EFCs to power implantable devices.

## Conclusions and Outlook

In addition to being a method of green energy production, enzymatic fuel cells offer many advantages such as potential for miniaturization and flexibility of fuels. With proper engineering of various components of the EFC, scientific community has come a long way in enhancing the EFC performance including power density and open circuit potential in a push toward real-life applications, especially in the biomedical field. Both biocatalysts and electrodes were engineered to promote higher catalytic activity, more efficient electron transfer, and faster current collection to maximize power generation. Some engineering methods have been employed to prolong the activity of the biocatalysts and therefore the stability of the EFC performance.

Even though many potential applications have been explored, some of which were very promising, EFCs still suffer greatly from lack of long-term stability and low power density with fuel concentrations as low as physiological conditions. However, the ongoing debate on whether or not MET is better than DET or vice versa may be the bottleneck that takes away the focus from realization of EFCs in real-life applications. More research based on fundamentals of enzymatic and electroactivity of the biocatalysts may be necessary to undeniably support or refute the direct electron transfer of some popular enzymes like glucose oxidase.

## Author Contributions

SY prepared the manuscript and obtained permission to reuse figures from appropriate parties. All authors contributed to the article and approved the submitted version.

## Conflict of Interest

The authors declare that the research was conducted in the absence of any commercial or financial relationships that could be construed as a potential conflict of interest.
